# Antimicrobial Terpenes Suppressed the Infection Process of *Phytophthora* in Fennel-Pepper Intercropping System

**DOI:** 10.3389/fpls.2022.890534

**Published:** 2022-06-09

**Authors:** Yuxin Yang, Ying Li, Xinyue Mei, Min Yang, Huichuan Huang, Fei Du, Jiaqing Wu, Yiyi He, Junwei Sun, Haining Wang, Xiahong He, Shusheng Zhu, Yingbin Li, Yixiang Liu

**Affiliations:** ^1^State Key Laboratory for Conservation and Utilization of Bio-Resources in Yunnan, Yunnan Agricultural University, Kunming, China; ^2^Key Laboratory for Agro-Biodiversity and Pest Control of Ministry of Education, College of Plant Protection, Yunnan Agricultural University, Kunming, China; ^3^China France Plantomix Joint Laboratory, Yunnan Agricultural University, Kunming, China

**Keywords:** synergistic antimicrobial ability, intercropping, *Phytophthora capsici*, terpene compounds, reactive oxygen species

## Abstract

The interactions between non-host roots and pathogens may be key to the inhibition of soilborne pathogens in intercropping systems. Fennel (*Foeniculum vulgare*) can be intercropped with a wide range of other plants to inhibit soilborne pathogens in biodiversity cultivation. However, the key compounds of fennel root exudates involved in the interactions between fennel roots and pathogens are still unknown. Here, a greenhouse experiment confirmed that intercropping with fennel suppressed pepper (*Capsicum annuum*) blight disease caused by *Phytophthora capsici*. Experimentally, the roots and root exudates of fennel can effectively interfere with the infection process of *P*. *capsici* at rhizosphere soil concentrations by attracting zoospores and inhibiting the motility of the zoospores and germination of the cystospores. Five terpene compounds (D-limonene, estragole, anethole, gamma-terpenes, and beta-myrcene) that were identified in the fennel rhizosphere soil and root exudates were found to interfere with *P. capsica* infection. D-limonene was associated with positive chemotaxis with zoospores, and a mixture of the five terpene compounds showed a strong synergistic effect on the infection process of *P*. *capsici*, especially for zoospore rupture. Furthermore, the five terpene compounds can induce the accumulation of reactive oxygen species (ROS), especially anethole, in hyphae. ROS accumulation may be one of the antimicrobial mechanisms of terpene compounds. Above all, we proposed that terpene compounds secreted from fennel root play a key role in *Phytophthora* disease suppression in this intercropping system.

## Introduction

Crop diversity is a global trend (Keesing and Ostfeld, 2015), and it has been confirmed that increasing plant diversity in ecosystems not only enhances stability but also prevents biological stresses (Flombaum and Sala, 2008; Newton et al., 2009). Compared with monoculture, biodiversity intercropping can improve utilization of land (Homulle et al., 2021) and enhance nitrogen absorption to increase yield (Gómez-Rodrìguez et al., 2003), especially for disease management in the field (Zhu et al., 2000). For example, cereal/fava bean (*Vicia faba* L) mixtures decrease the disease incidence of yellow rust and mildew in wheat (Zhang et al., 2019), and cotton (*Gossypium spp*)/cucumber (*Cucumis sativus* L.) intercropping can suppress cotton root rot and wilt (Armanious, 2000). Maize (*Zea mays* L) grown between pepper (*Capsicum annuum*) rows reduced the levels of *Phytophthora* blight of pepper (Yang et al., 2014), and rice-water spinach (*Spinacia oleracea* L.) intercropping effectively controlled rice sheath blight and leaf folders (Ning et al., 2017).

Root exudates of intercropped species can protect neighboring crop plants by directly inhibiting spore germination and mycelial growth, thus reducing pathogen populations in the soil (Zhu and Morel, 2019). *Phytophthora* (pepper blight) is one of the most wide spread and devastating soil-borne pathogens of pepper (Hwang and Kim, 1995). Pepper intercropping with other crops is an effective and economic measure to suppress the spread of this pathogen in the soil (Gao et al., 2014; Yang et al., 2014). Non-host maize plant roots can form a “root wall” below the ground that attracts zoospores of *P*. *capsici* and simultaneously secretes antimicrobial compounds to kill zoospores, similar examples include garlic (*Allium sativum* L.) root against *P*. *capsica* (Liao et al., 2015), maize root against *Phytophthora sojae* and rape (*Brassica napus* L.) root against *Phytophthora nicotiana* (Gao et al., 2014; Fang et al., 2016). In addition, the rotation of fennel (*Foeniculum vulgare*) with tobacco (*Nicotiana tabacum* L.) suppressed tobacco black shank caused by *Phytophthora parasitica* (Zhang, 2012). However, there are limited data on the underlying mechanisms by which fennel roots interfere with *Phytophthora* infestation in diversity systems.

Root exudates, including flavone phenolic alcohols, terpenes, isothiocyanates, and glucosinolates (Ren et al., 2003; Kliebenstein, 2009; Wu et al., 2009; Sanchez-Vallet et al., 2010; Cruz et al., 2012; Liu H. et al., 2021), improved plant growth and weakened soil-borne pathogens. Among those compounds, terpenes are hydrocarbons that contain one or more carbon–carbon double bonds and share the same elementary unit of isoprene (2-methyl-1,4-butadiene), with additional pollinator-attractive properties and strong antimicrobial activity against microorganisms (Dalleau et al., 2008; Pandey et al., 2014). Thus, we hypothesized that terpenes may be the key component of the interaction between non-host plant roots and pathogens, and their underlying mechanisms remain to be further studied.

Reactive oxygen species (ROS) are considered to be important in signal transduction pathways in the interaction between plants and pathogens (Voges et al., 2019). Under normal conditions, ROS production and ROS elimination are balanced; however, oxidative stress represents an imbalance due to an increase in ROS (Sies, 2018). Previous studies have found that oxidative stress may play an important role in the antimicrobial mechanisms of natural active products of plants, such as cinnamaldehyde and vanillin, which can inhibit hyphal growth and induce ROS accumulation in *P*. *nicotianae* (Scott and Eaton, 2008; Torres, 2010; Finkel, 2011; Mittler et al., 2011; Yang et al., 2021).

The aims of this study were to use fennel/pepper*-P. capsici* as a model (1) to study fennel and pepper intercropping for disease suppression in a greenhouse; (2) to observe the interaction between fennel roots and *P. capsica* and identify the terpene compounds in root exudates; and (3) to determine the accumulation of ROS in *P*. *capsici* hyphae and illustrate the antimicrobial mechanism to reveal the potential mechanism of infection behavior suppression in *P*. *capsici* blight by non-host plants.

## Materials and Methods

### Plants and Pathogen

The pepper (cultivar “Tianjiao 6”) and fennel (cultivar “Siji”) seeds, and *Phytophthora capsici* (strain 501) were provided by the State Key Laboratory for Conservation and Utilization of Bio-Resources, Yunnan Agricultural University. *P*. *capsici* was cultured on V8 medium (30 mL of V8 juice, 6.0 g of agar, 0.5 g of CaCO_3_, 300 mL of ddH_2_O, 121°C for 20 min) at 25°C under a 12-h light-dark cycle for 8 days to produce sporangia. Then, 15 mL of sterilized water was added to the plate and placed in a 4°C refrigerator for 30 min before returning to room temperature for 30 min to release zoospores. The zoospore suspension was collected after filtration with gauze (8 m × 0.84 m) and diluted to a concentration of 10^5^ zoospores/mL for further use.

### Greenhouse Assay

**FIGURE 1:**
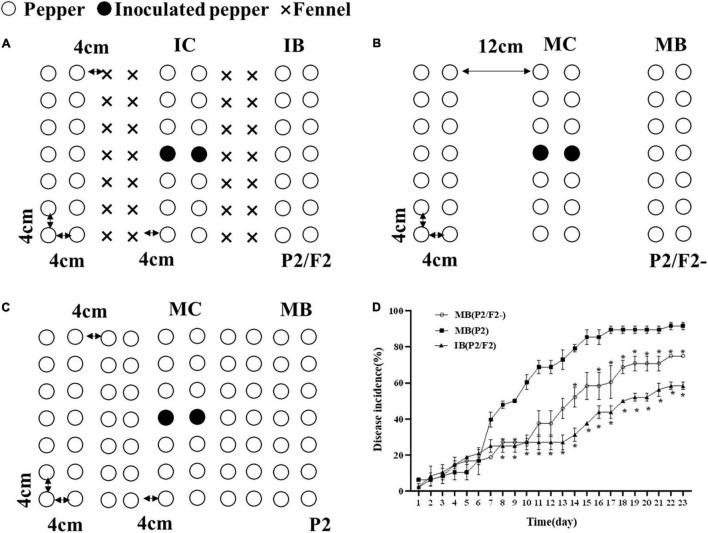
Pepper and fennel intercropping patterns and their effects on pepper *Phytophthora* blight control in the field. **(A)** Pepper and fennel intercropping; **(B)** pepper/blank intercropping; **(C)** pepper monoculture; **(D)** disease incidence of *Phytophthora* pepper blight in monoculture systems, intercropping systems and the intercropping spacing without fennel rows. IB and MB indicate the borderlines (indicator line) in the intercropping and monoculture fields, respectively. IC and MC indicate the centerlines (inoculate online) in the intercropping and monoculture fields, respectively. Significant differences are based on Chi square test (*p* < 0.05). The error bars indicate the standard errors of the means (*n* = 3). The significant differences were compared between monoculture (P2), intercropping with fennel (P2/F2), and peppers spaced as in intercropping but without fennel (P2/F2-). *Indicate *p* < 0.05, respectively.

To determine the effect of the pepper and fennel intercropping system on *Phytophthora* pepper disease suppression, greenhouse experiments were designed. Pepper and fennel seeds were surface sterilized with 3% sodium hypochlorite and then pregerminated in a humid chamber for 3 days at 25°C in the dark. Pepper seeds were sown after the fennel seeds were planted for 7 days in a plastic basin (50 by 30 cm). The greenhouse temperature range was approximately 25–30°C. To confirm the role of fennel plants in the intercropping system, pepper/fennel intercropping was performed (P2/F2), as shown in Figure 1A. In the second experiment, all pepper plants were planted with the same row spacing (P2/F2-) to eliminate the influence of physical distance (Figure 1B). In the third experiment, a pepper monoculture (P2) was designed (Figure 1C). Each treatment was replicated three times. One milliliter of a zoospore suspension (10^5^ mL^–1^) of *P*. *capsici* was injected into the central pepper seedling in the central pepper row after 30 days (seeding stage). Intercropping borderlines (IB) and monoculture borderlines (MB) indicate the borderlines (indicator line) in the intercropping and monoculture fields, respectively. Intercropping centerlines (IC) and monoculture centerlines (MC) indicate the centerlines (inoculate online) in intercropping and monoculture fields, respectively. Disease incidence in inoculation and indicator rows was investigated from 3 dpi (days postinoculation) until all pepper plants in the inoculation rows were dead.

Disease incidence rate of pepper border row = 100 × [Number of infected plants in border row/Total number of investigated plants in border row]

### Field Experiment

A field trial was conducted in 2021 at the Xundian County, Kunming City (N25°20′, E102°41′), Yunnan Province, China. To determine the effect of the pepper and fennel intercropping system on pepper *Phytophthora* blight suppression, we conducted field experiments using a single factor randomized block design with three replicates. And they include pepper monoculture and removing fennel rows from the intercropping system and pepper/fennel intercropping. Each treatment was replicated three times. All the plots were located in the same field and arranged using a randomized block design. When the pepper plants had grown to the fruit period, Phytophthora capsicum cake was used to inoculate the pepper. In brief, the pepper plants were cut with a blade at 0.5 cm below the stem base, and then the cake was put in the wound. The incidence rates of pepper *Phytophthora* disease in the center and border rows were surveyed to show the ability to spread in and across rows. The incidence was calculated by using the following formula.

Disease incidence rate of pepper in the center row = Number of infected plants in center row/Total number of investigated plants in center row × 100%.

Disease incidence rate of pepper border row = Number of infected plants in border row/Total number of investigated plants in border row × 100%.

### The Interaction Between Fennel Root and Zoospores of *P. capsici*

A special apparatus was used to monitor the interaction between the spores of *P*. *capsici* and fennel roots (Yang et al., 2014). Briefly, a U-shaped chamber was formed by placing a bent capillary tube on a glass slide and covering this with a coverslip. The root cap side of fennel roots was inserted into a zoospore suspension (10^5^ spores mL^–1^) in the chamber. The behavior of the zoospores in the root cap was recorded every 5 min for a period of 30 min by taking five photographs of each zone (Olympus BX43F, Japan). A capillary tube replaced the root as the control. The numbers of zoospores, cystospores and ruptured cystospores in the different root zones were counted. The chemotactic ratio (CR) was calculated as “scores of the zoospores and cystospores on the test root” divided by “scores of the zoospores and cystospores in the control” (Halsall, 1976). CR > 1 indicated positive chemotactic activity. Each interaction assay was replicated three times. Then, the inhibition ratio of swimming zoospores and cystospore germination were calculated (Zhang et al., 2020).

Ratio of zoospore rupture (%) = (Number of ruptured zoospores)/(Number of total zoospores) × 100.

### Collection of Fennel Root Exudates

Fennel plants were cultured using a previously described method (Yang et al., 2014), and root exudates were collected by the water culture method (Yuan et al., 2015). The fennel seeds were sown in black plastic pots with 40% humus soil and 60% field soil (poor soil outside the planting area) and grown for 45 days. The collected liquids were filtered, extracted twice with ethyl acetate, and concentrated under reduced pressure (Rotavapor R-200, Buchi). Finally, the concentrate was weighed, redissolved in 1 mL of methanol and filtered through a 0.22 μm filter. The concentration of the fennel root exudate stock was 10,000 μg/mL and that of the collected fluids before concentration was 25 μg/mL. The root exudates were prepared for antimicrobial assays.

### Identification of Terpene Compounds in Fennel Root

Headspace solid-phase microextraction (HS-SPME) of fennel root volatiles was conducted. The volatiles were collected from fennel roots that had been growing for 45 days. First, 20 fennel roots were put into the collection flask, and the SPME head was inserted at the top of the collection flask and collected at 25°C for 1 h (Li et al., 2018). Then, the head of extraction for collecting volatiles was directly inserted into the thermal analytical injection port for gas chromatography–mass spectrometry (GC–MS) analysis. The GC–MS fingerprints of the root volatiles were obtained on a GC–MS instrument (Shimadzu QP2010, Japan) (Block, 2010). Then, fennel root volatiles were separated on an SH-Rxi-5Sil MS capillary column (221-75954-30, 30 m × 0.25 mm × 0.25 μm, Shimadzu, Japan). The pressure was maintained at 49.5 kPa, giving a column flow of 1 mL/min. The injection volume was 1 μL in split-less mode, and the injector temperature was 250°C. The initial column temperature was 40°C (hold 2 min) and programmed to increase at a rate of 3°C/min to 80°C and then continue increasing to 270°C at a rate of 5°C/min, where it was then held for 10 min. The ion source temperature was 230°C with an interface temperature of 250°C. Helium (99.999% purity) was used as the carrier gas at a flow rate of 1 mL/min. Mass spectra were obtained in electron impact (EI) ionization mode at 70 eV by monitoring the full-scan range (m/z 35-500). The compounds were identified by matching the obtained mass spectra with those of reference compounds stored in the NIST14 library, except for the compounds that appeared in the control. Then, the standards of terpene compounds were analyzed by GC–MS under similar conditions (Zhang et al., 2020).

### Identification of Terpene Compounds in Fennel Rhizosphere Soil

The collection of rhizosphere soil was based on the methods of Bai et al. (2015) with slight modifications. The collected soil (with cultured fennel) and control soil (without any plants) were dried in an oven at 60°C for 12 h. At this time, the water content of the collected soil was 43.02%, and the water content of the control soil was 33.4%. Based on this result, the content of terpenes in the soil water could be calculated.

Experiments were designed to obtain the final concentration range of terpene compounds in fennel rhizosphere terpene standards with different concentrations (0, 1, 10, 20, 30, 100 μg/mL) were added to the blank dry soil (B-soil) to reach the same soil water content as the rhizosphere soil of fennel, and the method of collecting B-soil volatiles and determining the GC–MS fingerprint were the same as those described for fennel root exudates. The regression equation between the terpene standards with different concentrations and the GC–MS fingerprints of the fennel rhizosphere soil were obtained. The concentration-peak area standard curve was used to obtain the contents of terpenes in the soil sample to be measured.

### Antimicrobial Activity of Fennel Root Exudates Against Zoospore Motility and Cystospore Germination

The fennel root exudate stock (10,000 μg/mL) was diluted 2, 5, 10, 20, 50, and 100 times, and distilled water containing the same concentration of methanol was used as a control treatment. The chemotaxis of fennel root exudates on zoospores of *P. capsica* was observed using a previously reported method (Fang et al., 2016). A square groove was made with a capillary of 1 mm in diameter, and then the square groove was placed on a glass slide (length 25 mm × width 25 mm × height 1 mm). Zoospore suspension at a concentration of 10^5^ spores/mL was added to the tank. One end was treated with a capillary tube to which a diluted root exudate was added, and a capillary containing the same concentration of methanol at one end was used as a control. The behavior of zoospores was observed under a microscope with 40-fold magnification. The experiment was performed three times, each time in triplicate.

The zoospore motility and cystospore germination effects of the root exudates on *P. capsici* were tested as described by Yang et al. (2014). Briefly, 5 μL of root exudates and 20 μL of zoospore suspension (10^5^ zoospores/mL) or cystospore suspension (10^5^ cystospores/mL) were immediately mixed in glass slides. Then, the final concentration of the root exudates was diluted 5, 10, 25, 100, 150, and 250 times, and the concentrations reached 100, 50, 20, 10, 5, and 2 μg/mL in the slide. The slides were placed in Petri dishes containing moist filter paper and incubated at 25°C in the dark.

Photographs of immobilized zoospores and germinated cystospores were taken under a light microscope. Then, the percentage of zoospores encysted into cystospores was recorded every 1 min for a period of 5 min, and the percentage of germinated-cystospores was calculated after incubation for 1.5 h. Three fields were observed in each treatment, with 100–150 spores per field and three replicates per treatment.

### Antimicrobial Activity of Terpene Compounds Against *P*. *capsici*

Based on actual concentrations of the rhizosphere measured by GC–MS, the antimicrobial effect of the terpene compounds from the fennel exudates on the infection behavior of *P*. *capsici* (chemotaxis, zoospore motility, cystospore germination, and hyphal growth) was determined. The terpene compounds were prepared as 1 × and 2 × mixed terpene compounds (MTCs) according to their concentration proportions in the rhizosphere soil of fennel, and each single terpene compound at different concentrations was tested separately using the method with a few modifications described below (Miller et al., 1983).

Six mycelial plugs (7 mm in diameter) of *P*. *capsici* were placed in 100 mL flasks containing 60 mL of carrot liquid medium and cultured for 36 h at 28°C and 140 rpm. Then, 600 μL of terpene compound solutions of different concentrations were added to each flask, and 600 μL of methanol solution without terpene compound was added as the control; each treatment was repeated four times. After continuous culturing for 12 h, the liquid medium was removed by filtration; the hyphae were then wrapped in filter paper, dried and weighed to calculate the inhibition rate of the hyphae. The calculation method was as follows: mycelium inhibition rate (%) = (mycelia weight of control 1- treated mycelia weight)/(mycelia weight of control 1- mycelia weight of control 2) × 100. Control 1 was the mycelial weight after treatment with 600 μL of methanol solution without terpene compounds, and control 2 was the mycelial weight after culturing for 36 h at 28°C and 140 rpm.

### Measurement of Intracellular Reactive Oxygen Species

Intracellular ROS generation was monitored by fluorescence microscopy using a 2′,7′-dichlorodihydrofluorescein diacetate (H_2_DCFDA) fluorescent probe. The non-fluorescent compound can be oxidized by cellular oxidants into the highly fluorescent compound 2′,7′-dichlorofluorescin (DCF), which is trapped inside the cells (Murphy et al., 2011). Thus, the fluorescence intensity is proportional to the level of peroxide produced by the cells. Briefly, the mycelium was washed with PBS and then stained with 10 μM H_2_DCFDA at 25°C for 30 min after treatment with fennel root exudates and terpene standard compounds for 24 h (Xiao et al., 2013). The fluorescence of DCF was detected by an inverted fluorescence microscope (Leica DM 2000, Germany).

### Data Analysis

Statistical analysis was performed with SPSS statistics 24.0 (Stanford University, Stanford, CA, United States) using Chi square test, one-way ANOVA and Duncan’s multiple comparisons test, and figures were generated by GraphPad Prism 8 for Windows 10 (Microsoft Corporation, Redmond, WA, United States).

## Results

### Effect of Fennel and Pepper Intercropping on *P. capsici* Disease Control

As Figure 1 shows, the intercropping of fennel and pepper (P2/F2) significantly decreased pepper *Phytophthora* blight in the indicator rows (*p* < 0.05) (Figures 1A–C) compared with pepper monoculture (P2). Moreover, even though the pepper plants in the monoculture (P2/F2-) were planted with the same row spacing used in intercropping (Figure 1B), the disease incidence in the indicator row of the intercropping group was significantly lower than that in the monoculture, and the incidence of IB (P2/F2), MB (P2/F2-) and MB (P2) was 58%, 75%, 91.67%, respectively (Figure 1D), during the 23-day observation period. Therefore, the intercropping of fennel and pepper could significantly inhibit the occurrence and spread of *Phytophthora* pepper blight.

### Fennel Roots Interfere With the Infection Behavior of Zoospores

**FIGURE 2:**
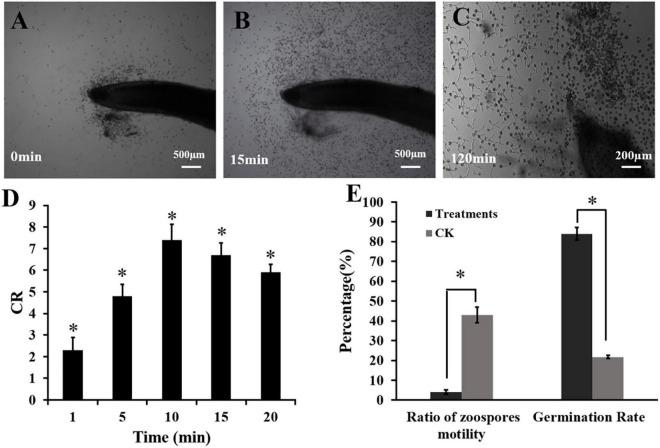
Effects of fennel roots on zoospore behavior of *P. capsici.* **(A,B)** Dynamic process of *P. capsica* zoospore attraction by fennel roots. **(C)** Cystospore germination. **(D)** Chemotaxis ratios (CR): fennel root showed significant attraction to zoospores. **(E)** Germination rate and motility rate in fennel root and zoospore interactions. CK was a capillary tube replacing the root as the control. Significant differences were based on Chi square test (*p* < 0.05). The error bars indicate the standard error of the means (*n* = 3). *Indicates significant differences between the control and plant root treatment at the 0.05 level.

The interaction between the fennel roots and *P*. *capsici* spores was observed under a microscope. As shown in Figure 2, zoospores of *P*. *capsici* showed strong chemotaxis to fennel root (Figure 2A). The dynamic chemotactic process showed that zoospores were significantly attracted by fennel root in 5 min, with chemotaxis ratios (CR) of 4.8 (Figure 2D). After approaching the fennel roots, the zoospores rapidly lost their swimming ability and transformed into cystospores, and the ratio of zoospore motility was only 4.14% (Figures 2B,E). After 30 min of incubation, all zoospores stopped swimming compared with those in the control treatment. After 120 min, fennel roots effectively promoted the germination of cystospores, which was 62.23% higher than that of the control (Figures 2C,E).

### Fennel Root Exudates Interfere With the Infection Behavior of Spores

**FIGURE 3:**
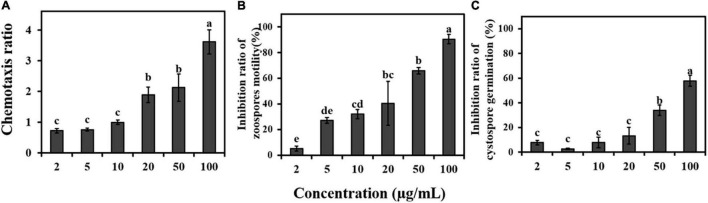
Effects of fennel root exudates on zoospores at different stages. **(A)** Chemotaxis. **(B)** Motility. **(C)** Cystospore germination. Significant differences are based on one-way ANOVA. The error bars indicate the standard error of the means (*n* = 3). Lower case letters show significant differences in the inhibitory effect at different stages at the 0.05 level.

The fennel root exudates showed a strong ability to attract zoospores of *P*. *capsici* in a 20 μg/mL methanol solution, and the chemotaxis index of *P*. *capsici* was positively correlated with the concentration of root exudates (Figure 3A). Fennel root exudates inhibited the motility of zoospores and germination of cystospores in a dose-dependent manner. When the concentration of root exudates reached 20 μg/mL (close to the concentration of the root exudates before concentration), the inhibition rates for zoospore motility and cystospore germination were 40.5% and 13.2%, respectively (Figures 3B,C).

### Compounds Identified in Fennel Rhizosphere Soil

**FIGURE 4:**
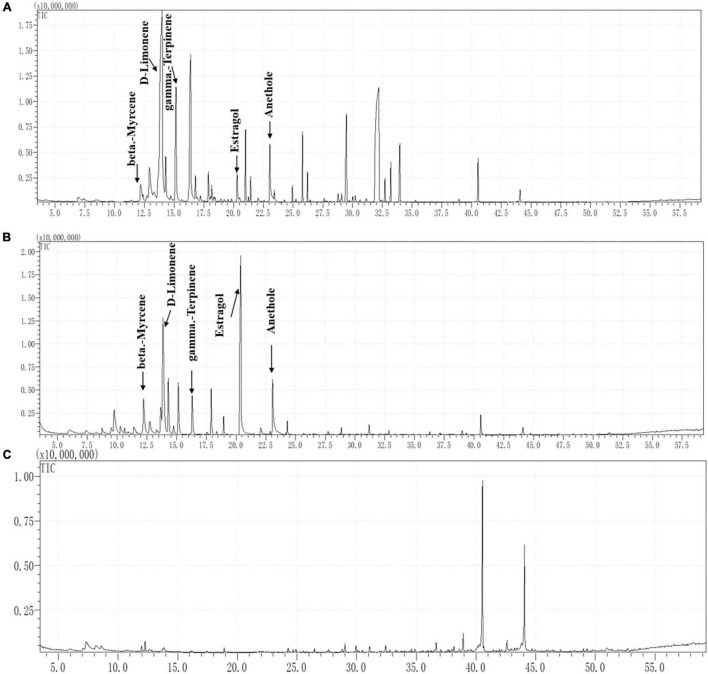
Separation and characterization of terpene compounds from fennel root volatiles and fennel rhizosphere soil by gas chromatography (GC) mass spectrometry (MS) analysis. **(A)** GC–MS profiles of root volatiles, **(B)** GC–MS profiles of rhizosphere soil, and **(C)** GC–MS profiles of the control soil.

As Figure 4 shows, the GC–MS profiles of fennel root volatiles and rhizosphere soil showed five peaks at the following retention times (RT): 12.25 min (beta-myrcene), 13.80 min (D-limonene), 16.30 min (gamma-terpinene), 20.25 min (estragole), and 23.10 min (anethole) (Figures 4A,B). The characteristic fragment of terpene compounds showed more than 90% similarity in root exudates or rhizosphere soil compared with the standards of terpene compounds. However, none of the five terpenes were detected in the control soil (Figure 4C). The contents of D-limonene, estragole, anethole, gamma-terpinene and beta-myrcene were 67.48, 13.54, 18.30, 66.30 and 6.13 μg/g, respectively, in fennel rhizosphere soil. Quantitative analysis of terpenes in rhizosphere soil showed that the content of D-limonene in the rhizosphere soil of fennel was the highest (67.48 ± 12.99 μg/g), and the content of beta-myrcene was the lowest (6.13 ± 0.70 μg/g) (Table 1).

**TABLE 1:** Quantitative analysis of terpenes in rhizosphere soil.

Analyte	Retention time (RT) (min)	Molecular weight	Calibration curve	R^2^	Actual concentration (μg/g)
Beta-Myrcene	12.33	136.23	y = 313474x – 1E + 06	0.9958	6.13 ± 0.70
D-limonene	14.02	136.23	y = 82102x + 648887	0.9973	67.48 ± 12.99
gamma-Terpinene	15.26	136.23	y = 27272x + 26867	0.9992	66.30 ± 0.96
Estragole	20.43	148.2	y = 425864x – 4E + 06	0.9989	13.54 ± 0.71
Anethole	23.22	148.2	y = 217035x – 86524	0.9941	18.30 ± 3.38

*Control, soil without plant culture. The error bar indicates the standard errors of the means (n = 3).*

### Terpene Compounds Interfere With the Behavior of *P*. *capsici*

**FIGURE 5:**
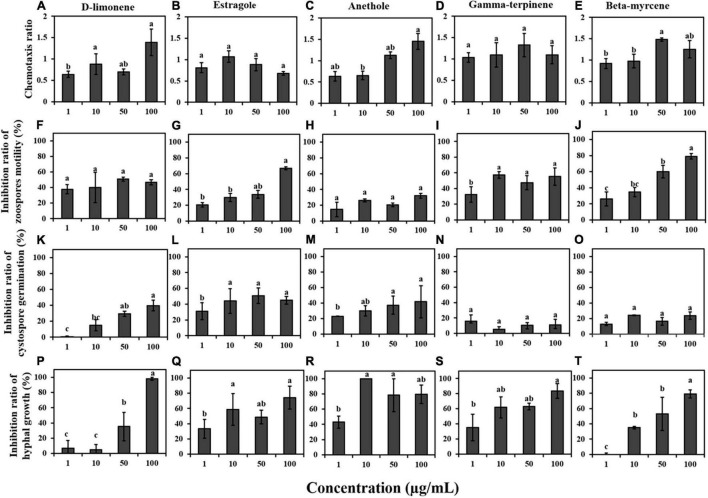
**(A–E)** Chemotaxis of terpene compounds on zoospores. **(F–J)** Inhibition of terpene compounds on zoospore motility of *P*. *capsici*. **(K–O)** Inhibition of terpene compounds on cystospore germination. **(P–T)** Inhibition of terpene compounds on hyphal growth. Significant differences are based on one-way ANOVA (*p* < 0.05). The error bars indicate the standard errors of the means (*n* = 3).

As shown in Figures 5A–E, the CR of each compound exhibited the ability to attract zoospores of *P*. *capsici* to a certain extent. Among the five terpenes, D-limonene (100 μg/mL), estragole (10 μg/mL), and anethole (50 and 100 μg/mL) were associated with positive chemotaxis, and gamma-terpinene and beta-myrcene showed chemotaxis ratios close to 1 at 1, 10, 50, and 100 μg/mL. However, the five terpenes had strong inhibitory effects on the motility of *P*. *capsici* zoospores at 100 μg/mL. The inhibition ratios of swimming ability for each of the five terpenes were 50.88, 66.83, 32.16, 55.27, and 79.05% at 100 μg/mL, respectively (Figures 5F–J). In addition, D-limonene and anethole inhibited cystospore germination in *P*. *capsici*; the inhibition ratio was the highest at a concentration of 100 μg/mL; and estragole showed strong activity against cystospore germination at concentrations of 1, 10, 50, and 100 μg/mL (Figures 5K–O). These five terpenes significantly inhibited the mycelial growth of *P. capsici* within the effective concentration range, and the inhibition ratio reached 96.5% at 10 μg/mL anethole. Except for anethole and estragole, the higher the concentration of the other three terpene compounds was, the higher the mycelial inhibition (Figures 5P–T).

To further explore the effect of terpenes on the behavior of *P*. *capsici*. The five terpene compounds were mixed according to their concentration proportions in the rhizosphere soil of fennel. As shown in Table 2, the five terpene compounds were not attractive to zoospores, and the terpene mixture was slightly more attractive than its corresponding single compound. In contrast to the ability of the terpene mixtures and D-limonene to attract *P*. *capsica* zoospores, the other four terpene compounds could not attract zoospores at twice the rhizosphere concentration. The inhibitory effect of the mixture on zoospore motility was significantly higher than that of any terpene compound alone, and both 1 × and 2 × MTC reached 100% (Table 2). Analysis of the inhibitory effect of the terpene mixtures on cystospore germination also showed that 2 × MTC had a 19.08% stronger effect than 1 × MTC, which was significantly higher than that of any terpene compound alone (Table 2). Regarding the inhibition of hyphal growth, estragole and anethole showed similar activity against hyphal growth and exhibited no significant difference from the terpene mixtures. However, the terpene mixtures of 1 × and 2 × MTC showed the highest ratios of zoospore rupture, 89.43% and 98.85%, respectively, compared to a corresponding single compound.

**TABLE 2:** The antimicrobial activity of a mixture of five terpene compounds against *Phytophthora capsici* infection according to their concentrations in the rhizosphere soil of fennel.

			Inhibition ratio (%)			
			
Concentration	Compound	Chemotaxis	Zoospore motility	Cystospore germination	Hyphal growth	Ratio of zoospore rupture (%)
× 1 (1 time)	D-limonene	0.71 ± 0.12a	45.86 ± 1.23b	21.85 ± 9.22c	27.22 ± 4.12a	4.67 ± 1.62b
	Estragole	0.85 ± 0.08a	36.79 ± 3.50c	28.51 ± 1.72b	30.32 ± 5.90a	4.89 ± 2.00b
	Anethole	0.57 ± 0.14a	41.69 ± 1.63b	41.14 ± 8.49ab	49.18 ± 9.73a	7.92 ± 1.28b
	gamma-Terpinene	0.95 ± 0.47a	44.42 ± 1.80b	28.01 ± 14.36ab	56.91 ± 5.14a	0.58 ± 0.66b
	beta-Myrcene	1.01 ± 0.40a	34.1 ± 2.18b	18.85 ± 2.06c	34.97 ± 12.05a	3.97 ± 1.16b
	1 × MTC	1.00 ± 0.35a	100 ± 0a	64.88 ± 9.41a	51.73 ± 1.11a	89.43 ± 1.32a
× 2 (2 times)	D-limonene	1.41 ± 0.23a	44.175 ± 1.18b	30.31 ± 5.38c	51.18 ± 7.11a	6.79 ± 2.46b
	Estragole	1.04 ± 0.36b	39.0 ± 1.91bcd	65.02 ± 8.00ab	64.59 ± 2.00a	1.84 ± 1.24b
	Anethole	0.36 ± 0.06ab	34.82 ± 1.03cd	46.26 ± 3.61bc	72.95 ± 8.84a	7.23 ± 3.27b
	gamma-Terpinene	0.86 ± 0.35ab	34.1 ± 2.38d	36.40 ± 2.99c	50.38 ± 6.82a	6.62 ± 2.65b
	beta-Myrcene	0.81 ± 0.29ab	40.8 ± 2.81bc	25.55 ± 3.57d	38.71 ± 8.30a	7.84 ± 2.84b
	2 × MTC	1.38 ± 0.17a	100 ± 0a	83.80 ± 11.46a	69.67 ± 14.11a	98.85 ± 0.94a

*1 × MTC and 2 × MTC indicate that the concentrations of the mixture were 1 and 2 times the rhizosphere soil concentrations, respectively (mixture = 6.13 μg/mL beta-myrcene + 67.48 μg/mL D-limonene + 66.30 μg/mL gamma-terpinene + 13.54 μg/mL estragole + 18.30 μg/mL anethole).*

*Different letters in the same group indicate significant differences as determined by one-way ANOVA (p < 0.05).*

*The error bars indicate the standard errors of the means (n = 3).*

### Fluorescence Detection of Reactive Oxygen Species Accumulation

**FIGURE 6:**
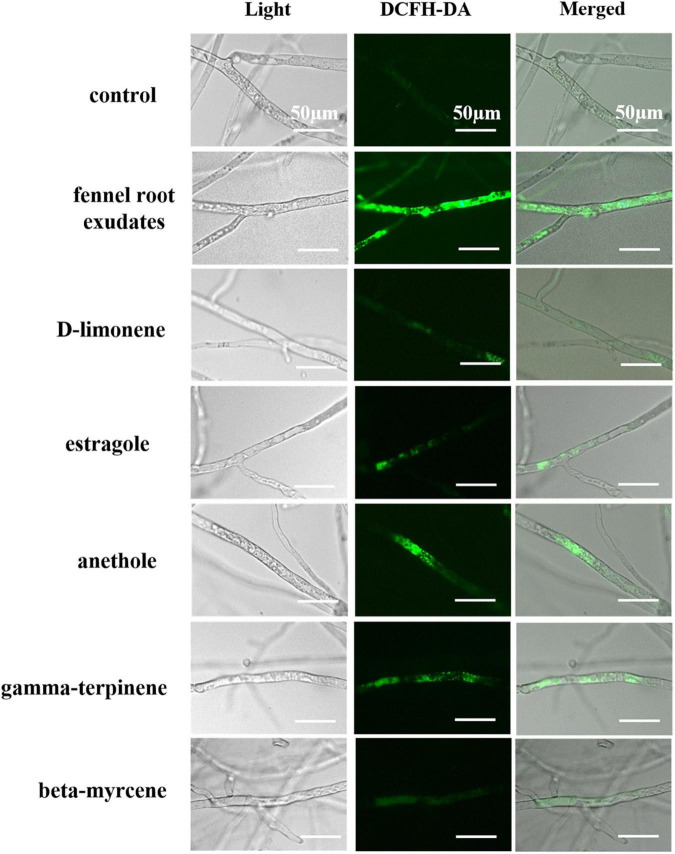
Fennel root exudates and terpene compounds induced ROS accumulation in *P. capsici* hyphae. Bar = 50 μm.

As determined by the DCHF-DA signal (Figure 6), fennel root exudates and the five terpene compounds (D-limonene, beta-myrcene, anethole, estragole, and gamma-terpene) significantly induced intracellular ROS accumulation, whereas almost no fluorescence was detected in the control group, suggesting that ROS accumulation may play a key role in hyphal growth inhibition or cell death.

## Discussion

In this study, intercropping fennel and pepper successfully prevented the spread of *P*. *capsici* and reduced the incidence of *Phytophthora* blight disease in the field (Figure 1). This intercropping model, using short rod-shaped fennel to control pepper blight, provides a new reference for spatial layout based on biodiversity theory compared with the intercropping pattern of “pepper blight control by high rod-shaped maize” (Yang et al., 2014). Furthermore, we found that the roots and root exudates of fennel could interfere with the infection process of *P. capsici* by attracting zoospores and then inhibiting motility (Figures 2, 3), which may play an important role in the inhibitory effect of fennel and pepper intercropping on pepper *phytophthora* blight. This hypothesis can be proven by previous research showing that some other non-host plants, such as maize and rapeseed, have also been reported to inhibit the swimming or germination of *Phytophthora* zoospores and suppress blight disease in the field (Fang et al., 2016; Zhang et al., 2020). However, the germination of cystospores was suppressed by root exudates in a dose-dependent manner but not by fennel roots (Figure 3), which may be related to the higher concentration of antimicrobial compounds in fennel root exudates.

Terpenes are the largest class of natural products and perform a variety of roles in mediating antagonistic and beneficial interactions among organisms (Gershenzon and Dudareva, 2007). In this research, five terpene compounds with the highest abundance were identified both in the fennel root volatiles and the fennel rhizosphere soil (Figure 4 and Table 1). Interestingly, D-limonene, gamma-terpinene, and beta-myrcene with the chemical formula C_10_H_16_ and estragole and anethole with the chemical formula C_10_H_12_O were isomers. Moreover, at the concentrations of these compounds in rhizosphere soil, only D-limonene showed significant ability to attract zoospores of *P. capsici*, similar to that of the mixture with five terpene compounds (Figure 5 and Tables 1, 2). These results indicated that D-limonene might be the main chemotactic substance in the terpene group that attracts zoospores of *P. capsici*. In addition, all five terpenes with concentrations in fennel rhizosphere soil could significantly inhibit the zoospore motility, cystospore germination and hyphal growth of *P. capsici.* However, estragole and anethole, which are aromatic oxygenated monoterpenes, showed greater suppression of cystospore germination and hyphal growth than monoterpenes, including D-limonene and beta-myrcene. These results indicated that benzene rings or oxygenated monoterpenes might improve the antimicrobial ability, which was consistent with previous reports that oxygenated monoterpenes exhibit better antibacterial activity than non-oxygenated monoterpenes (Campos-Requena et al., 2015; Guimaraes et al., 2019).

Chemotaxis is defined as oriented movement toward or away from a chemical stimulus (Bi and Sourjik, 2018), and we speculate that the taxis of *P. capsici* zoospores may be a complex process that consists of chemotaxis and electrotaxis. For example, high limonene doses exert an important signaling effect to attract the bacterium Xanthomonas citri subsp. citri and the fungus Penicillium digitatum (Rodríguez et al., 2011); the K^+^ concentration, bioconduction and electric fields (electrotaxis) share responsibility for this process (Ochiai et al., 2011; Galiana et al., 2019). Therefore, the mechanism of chemotaxis of terpene components still needs to be further investigated.

Synergistic antimicrobial reactions are common in the interaction between antimicrobial compounds and pathogens (Campos-Requena et al., 2015). This point supports our approach of performing an antimicrobial assay on mixtures of the five terpenes, which showed higher suppression of zoospore motility than the same concentrations of individual compounds (Table 2). More importantly, the terpene mixture could also cause rupture of almost all zoospores when individual compounds showed little effect (Table 2), indicating that the infection process of *P. capsici* was almost completely cut off by the synergistic interaction of terpenes in the fennel rhizosphere. This synergistic interaction could decrease the minimal effective concentration and increase antimicrobial activity against pathogens (Chung et al., 2011; Berditsch et al., 2015). Hence, the synergistic interaction of terpenes in the root rhizosphere may play a key role in the inhibitory effect of fennel/pepper intercropping on the spread of pepper phytophthora blight.

ROS accumulation has been proposed as the earliest event induced during small molecule substance-pathogen interactions (Camejo et al., 2016). Similarly, terpene compounds could induce ROS accumulation in hyphae, and terpenes with higher antimicrobial activity, such as anethole and gamma-terpinene exhibited a stronger ability to induce ROS accumulation (Figure 6), indicating that oxidative stress caused by terpenes may trigger the interference of fennel roots in the infection process of *P. capsici*. Previous studies have shown that allyl isothiocyanate induced ROS accumulation in *Fusarium solani* (Li et al., 2020). Liquiritin inhibited *P. capsici* mycelial growth and sporangial development and significantly induced H_2_O_2_ accumulation (Liu P. et al., 2021). Hence inducing ROS accumulation may be one of the key mechanisms widely employed by small molecule substances against pathogens.

## Conclusion

We used fennel roots and *P*. *capsici* as a model to research the mechanism underlying the inhibitory effect of fennel and pepper intercropping on *P*. *capsici.* The non-host plant (fennel) roots can attract zoospores of *P*. *capsici* and then secrete a series of antimicrobial compounds to kill the pathogen. Five antimicrobial compounds were identified in the fennel rhizosphere, and antimicrobial ability and synergistic interaction play key roles in the interference effect of fennel roots on the infection behavior of *P*. *capsici*. Moreover, ROS accumulation may be the most important mechanism of the inhibitory effect of key compounds in fennel on *P*. *capsici*.

## Data Availability Statement

The original contributions presented in this study are included in the article/Supplementary Material, further inquiries can be directed to the corresponding author/s.

## Author Contributions

YXL and YBL conceived the ideas and designed the methodology. YY, YL, and XM performed the field experiment. YY, JW, and HW performed the GC–MS experiments. JS and YH performed the HS-SPME experiments. YY, YL, and YM performed the biological activity testing of the standards. HH, FD, and SZ collected the data. YY, YL, and XH analyzed the data. YY, YL, YBL, and YXL wrote the manuscript. All authors contributed to the article and approved the submitted version.

## Conflict of Interest

The authors declare that the research was conducted in the absence of any commercial or financial relationships that could be construed as a potential conflict of interest.

## Publisher’s Note

All claims expressed in this article are solely those of the authors and do not necessarily represent those of their affiliated organizations, or those of the publisher, the editors and the reviewers. Any product that may be evaluated in this article, or claim that may be made by its manufacturer, is not guaranteed or endorsed by the publisher.

## References

[B1] Armanious A. H. (2000). *Studies on Some Cotton Diseases*. Doctoral Dissertation. Minya: Minia University.

[B2] Bai Y. Müller D. B. Srinivas G. Garrido-Oter R. Potthoff E. Rott M. (2015). Functional overlap of the *Arabidopsis* leaf and root microbiota. *Nature* 528 364–369. 10.1038/nature16192 26633631

[B3] Berditsch M. Jäger T. Strempel N. Schwartz T. Overhage J. Ulrich A. S. (2015). Synergistic effect of membrane-active peptides polymyxin b and gramicidins on multidrug-resistant strains and biofilms of *Pseudomonas aeruginosa*. *Antimicrob. Agents Chemother.* 59 5288–5296. 10.1128/AAC.00682-15 26077259 PMC4538509

[B4] Bi S. Sourjik V. (2018). Stimulus sensing and signal processing in bacterial chemotaxis. *Curr. Opin. Microbiol.* 45 22–29. 10.1016/j.mib.2018.02.002 29459288

[B5] Block E. (2010). *Garlic and Other Alliums*. Cambridge: Royal Society of Chemistry.

[B6] Camejo D. Guzmán-Cedeño Á Moreno A. (2016). Reactive oxygen species, essential molecules, during plant–pathogen interactions. *Plant Physiol. Biochem.* 103 10–23. 10.1016/j.plaphy.2016.02.035 26950921

[B7] Campos-Requena V. H. Rivas B. L. Pérez M. A. Figueroa C. R. Sanfuentes E. A. (2015). The synergistic antimicrobial effect of carvacrol and thymol in clay/polymer nanocomposite films over strawberry gray mold. *LWT Food Sci. Technol.* 64 390–396. 10.1016/j.lwt.2015.06.006

[B8] Chung P. Y. Navaratnam P. Chung L. Y. (2011). Synergistic antimicrobial activity between pentacyclic triterpenoids and antibiotics against *Staphylococcus aureus*, strains. *Ann. Clin. Microbiol. Antimicrob.* 10 25–31. 10.1186/1476-0711-10-25 21658242 PMC3127748

[B9] Cruz A. F. Hamel C. Yang C. Matsubara T. Gan Y. Singh A. K. (2012). Phytochemicals to suppress *Fusarium* head blight in wheat–chickpea rotation. *Phytochemistry* 78 72–80. 10.1016/j.phytochem.2012.03.003 22520499

[B10] Dalleau S. Cateau E. Bergès T. Berjeaud J.-M. Imbert C. (2008). In vitro activity of terpenes against *Candida* biofilms. *Int. J. Antimicrob. Agents* 31 572–576. 10.1016/j.ijantimicag.2008.01.028 18440786

[B11] Fang Y. Zhang L. Jiao Y. Liao J. Luo L. Ji S. (2016). Tobacco rotated with rapeseed for soil-borne *Phytophthora* pathogen biocontrol: mediated by rapeseed root exudates. *Front. Microbiol.* 7:894. 10.3389/fmicb.2016.00894 27379037 PMC4904020

[B12] Finkel T. (2011). Signal transduction by reactive oxygen species. *J. Cell Biol.* 194 7–15. 10.1083/jcb.201102095 21746850 PMC3135394

[B13] Flombaum P. Sala O. E. (2008). Higher effect of plant species diversity on productivity in natural than artificial ecosystems. *Proc. Natl. Acad. Sci. U.S.A.* 105 6087–6090. 10.1073/pnas.0704801105 18427124 PMC2329694

[B14] Galiana E. Cohen C. Thomen P. Etienne C. Noblin X. (2019). Guidance of zoospores by potassium gradient sensing mediates aggregation. *J. R. Soc. Interface* 16:20190367. 10.1098/rsif.2019.0367 31387479 PMC6731506

[B15] Gao X. Wu M. Xu R. Wang X. Pan R. Kim H. J. (2014). Root Interactions in a fennel/soybean intercropping system control soybean soil-borne disease, red crown rot. *PLoS One* 9:e95031. 10.1371/journal.pone.0095031 24810161 PMC4014482

[B16] Gershenzon J. Dudareva N. (2007). The function of terpene natural products in the natural world. *Nat. Chem. Biol.* 3 408–414. 10.1038/nchembio.2007.5 17576428

[B17] Gómez-Rodrìguez O. Zavaleta-Mejıìa E. González-Hernández V. A. Livera-Muñoz M. Cárdenas-Soriano E. (2003). Allelopathy and microclimatic modification of intercropping with marigold on tomato early blight disease development. *Field Crops Res.* 83 27–34. 10.1016/S0378-4290(03)00053-4

[B18] Guimaraes A. C. Meireles L. M. Lemos M. F. Guimaraes M. C. C. Endringer D. C. Fronza M. (2019). Antibacterial activity of terpenes and terpenoids present in essential oils. *Molecules* 24:2471. 10.3390/molecules24132471 31284397 PMC6651100

[B19] Halsall D. M. (1976). Zoospore chemotaxis in Australian isolates of *Phytophthora* species. *Can. J. Microbiol.* 22 409–422. 10.1139/m76-062 3276

[B20] Homulle Z. George T. S. Karley A. J. (2021). Root traits with team benefits: understanding belowground interactions in intercropping systems. *Plant Soil* 471, 1–26. 10.1007/s11104-021-05165-8

[B21] Hwang B. K. Kim C. H. (1995). Phytophthora blight of pepper and its control in Korea. *Plant Dis.* 79 221–227.

[B22] Keesing F. Ostfeld R. S. (2015). Is biodiversity good for your health? *Science* 349 235–236. 10.1126/science.aac7892 26185230

[B23] Kliebenstein D. J. (2009). A quantitative genetics and ecological model system: understanding the aliphatic glucosinolate biosynthetic network via QTLs. *Phytochem. Rev.* 8 243–254. 10.1007/s11101-008-9102-8

[B24] Li X. De Boer W. Ding C. Zhang T. Wang X. (2018). Suppression of soil-borne *Fusarium* pathogens of peanut by intercropping with the medicinal herb *Atractylodes lancea*. *Soil Biol. Biochem.* 116 120–130. 10.1016/j.soilbio.2017.09.029

[B25] Li Y. Liu Y. Zhang Z. Cao Y. Li J. Luo L. (2020). Allyl isothiocyanate (AITC) triggered toxicity and FsYvc1 (a STRPC family member) responded sense in *Fusarium solani*. *Front. Microbiol.* 11:870. 10.3389/fmicb.2020.00870 32477298 PMC7235336

[B26] Liao J.-J. Zhang L.-M. Zhang X.-D. Zheng X. Jiao Y.-G. He X.-X. (2015). Biological characteristics of similar root edge cells of Garlic and their inhibitory activity against *Phytophthora capsici*. *Plant Protect.* 41, 39–45. 10.3969/j.issn.0529-1542.2015.05.007

[B27] Liu H. Wu J. Su Y. Li Y. Zuo D. Liu H. (2021). Allyl isothiocyanate in the volatiles of *Brassica juncea* inhibits the growth of root rot pathogens of *Panax notoginseng* by inducing the accumulation of ROS. *J. Agric. Food Chem.* 69 13713–13723. 10.1021/acs.jafc.1c05225 34780155

[B28] Liu P. Cai Y. Zhang J. Wang R. Li B. Weng Q. (2021). Antifungal activity of liquiritin in *Phytophthora capsici* comprises not only membrane-damage-mediated autophagy, apoptosis, and Ca2+ reduction but also an induced defense responses in pepper. *Ecotoxicol. Environ. Saf.* 209:111813. 10.1016/j.ecoenv.2020.111813 33360212

[B29] Miller J. D. Taylor A. Greenhalgh R. (1983). Production of deoxynivalenol and related compounds in liquid culture by *Fusarium graminearum*. *Can. J. Microbiol.* 29 1171–1178. 10.1139/m83-179

[B30] Mittler R. Vanderauwera S. Suzuki N. Miller G. A. D. Tognetti V. B. Vandepoele K. (2011). ROS signaling: the new wave? *Trends Plant Sci.* 16 300–309. 10.1016/j.tplants.2011.03.007 21482172

[B31] Murphy M. P. Holmgren A. Larsson N.-G. Halliwell B. Chang C. J. Kalyanaraman B. (2011). Unraveling the biological roles of reactive oxygen species. *Cell Metab.* 13 361–366. 10.1016/j.cmet.2011.03.010 21459321 PMC4445605

[B32] Newton A. C. Begg G. S. Swanston J. S. (2009). Deployment of diversity for enhanced crop function. *Ann. Appl. Biol.* 154 309–322. 10.1111/j.1744-7348.2008.00303.x

[B33] Ning C. Qu J. He L. Yang R. Chen Q. Luo S. (2017). Improvement of yield, pest control and Si nutrition of rice by rice-water spinach intercropping. *Field Crops Res.* 208 34–43. 10.1016/j.fcr.2017.04.005

[B34] Ochiai N. DragIila M. I. Parke J. L. (2011). Pattern swimming of *Phytophthora citricola* zoospores: an example of microbial bioconvection. *Fungal Biol.* 115 228–235. 10.1016/j.funbio.2010.12.006 21354529

[B35] Pandey A. K. Mohan M. Singh P. Palni U. T. Tripathi N. N. (2014). Chemical composition, antibacterial and antioxidant activity of essential oil of *Eupatorium adenophorum* Spreng. from Eastern Uttar Pradesh, India. *Food Biosci.* 7 80–87. 10.1016/j.fbio.2014.06.001

[B36] Ren W. Qiao Z. Wang H. Zhu L. Zhang L. (2003). Flavonoids: promising anticancer agents. *Med. Res. Rev.* 23 519–534. 10.1002/med.10033 12710022

[B37] Rodríguez A. Andrés V. S. Cervera M. Redondo A. Alquézar B. Shimada T. (2011). The monoterpene limonene in orange peels attracts pests and microorganisms. *Plant Signal. Behav.* 6 1820–1823. 10.4161/psb.6.11.16980 22212123 PMC3329358

[B38] Sanchez-Vallet A. Ramos B. Bednarek P. López G. Piślewska-Bednarek M. Schulze-Lefert P. (2010). Tryptophan-derived secondary metabolites in *Arabidopsis thaliana* confer non-host resistance to necrotrophic *Plectosphaerella cucumerina* fungi. *Plant J.* 63 115–127. 10.1111/j.1365-313X.2010.04224.x 20408997

[B39] Scott B. Eaton C. J. (2008). Role of reactive oxygen species in fungal cellular differentiations. *Curr. Opin. Microbiol.* 11 488–493. 10.1016/j.mib.2008.10.008 18983937

[B40] Sies H. (2018). On the history of oxidative stress: concept and some aspects of current development. *Curr. Opin. Toxicol.* 7 122–126. 10.1016/j.cotox.2018.01.002

[B41] Torres M. A. (2010). ROS in biotic interactions. *Physiol. Plant.* 138 414–429. 10.1111/j.1399-3054.2009.01326.x 20002601

[B42] Voges M. J. Bai Y. Schulze-Lefert P. Sattely E. S. (2019). Plant-derived coumarins shape the composition of an *Arabidopsis* synthetic root microbiome. *Proc. Natl. Acad. Sci. U.S.A.* 116 12558–12565. 10.1073/pnas.1820691116 31152139 PMC6589675

[B43] Wu H.-S. Shen S.-H. Han J.-M. Liu Y.-D. Liu S.-D. (2009). The effect in vitro of exogenously applied p-hydroxybenzoic acid on *Fusarium oxysporum* f. sp. niveum. *Phytopathol. Mediterr.* 48 369–376.

[B44] Xiao H. Wang J. Yuan L. Xiao C. Wang Y. Liu X. (2013). Chicoric acid induces apoptosis in 3T3-L1 preadipocytes through ROS-mediated PI3K/Akt and MAPK signaling pathways. *J. Agric. Food Chem.* 61 1509–1520. 10.1021/jf3050268 23363008

[B45] Yang M. Zhang Y. Qi L. Mei X. Liao J. Ding X. (2014). Plant-plant-microbe mechanisms involved in soil-borne disease suppression on a maize and pepper intercropping system. *PLoS One* 9:e115052. 10.1371/journal.pone.0115052 25551554 PMC4281244

[B46] Yang Y. Zhang H. Fang Y. Li Y. Mei X. Hang H. (2021). Interference by non host plant roots and root exudates in the infection processes of *Phytophthora*. nicotianae. *Front. Agric. Sci. Eng.* 8:447–459. 10.15302/J-FAST-2021399

[B47] Yuan J. Zhang N. Huang Q. Raza W. Li R. Vivanco J. M. (2015). Organic acids from root exudates of banana help root colonization of PGPR strain *Bacillus amyloliquefaciens* NJN-6. *Sci. Rep.* 5:13438. 10.1038/srep13438 26299781 PMC4547103

[B48] Zhang C. Dong Y. Tang L. Zheng Y. Makowski D. Yu Y. (2019). Intercropping cereals with faba bean reduces plant disease incidence regardless of fertilizer input; a meta-analysis. *Eur. J. Plant Pathol.* 154 931–942. 10.1007/s10658-019-01711-4

[B49] Zhang D. Z. (2012). *Studies on the Effect of Rotation and Intercropping to Growth, Yield and Characteristics of Cured Tobacco Variety KRK26.* Ph.D. Dissertation. Changsha: Hunan Agricultural University.

[B50] Zhang H. Yang Y. Mei X. Li Y. Wu J. Li Y. (2020). Phenolic acids released in maize rhizosphere during maize-soybean intercropping inhibit *Phytophthora* blight of soybean. *Front. Plant Sci.* 11:886. 10.3389/fpls.2020.00886 32849668 PMC7399372

[B51] Zhu S. Morel J.-B. (2019). Molecular mechanisms underlying microbial disease control in intercropping. *Mol. Plant Microbe Interact.* 32 20–24. 10.1094/MPMI-03-18-0058-CR 29996677

[B52] Zhu Y. Chen H. Fan J. Wang Y. Li Y. Chen J. (2000). Genetic diversity and disease control in rice. *Nature* 406 718–722. 10.1038/35021046 10963595

