# Supplementary material for: Antimicrobial Terpenes Suppressed the Infection Process of Phytophthora in Fennel-Pepper Intercropping System
Source: Front Plant Sci. 2022 Jun 9;13:890534. doi: 10.3389/fpls.2022.890534 (PMC9218821; doi:10.3389/fpls.2022.890534)
Supplement: Supplementary file 1 [file Data_Sheet_1.DOCX]

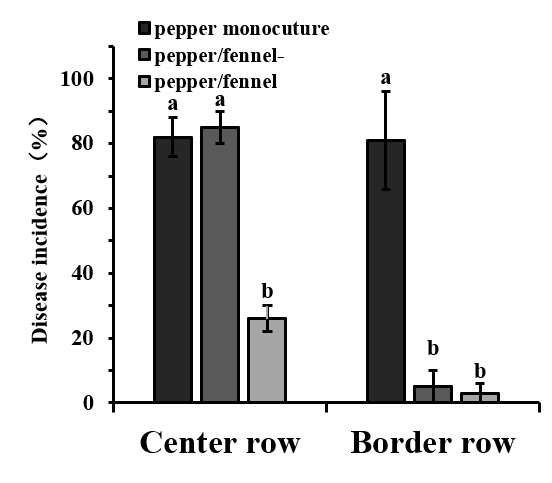


**FIGURE S1|** Disease incidence of pepper Phytophthora blight in monoculture and intercropping systems. IB and MB indicate the border lines (indicator line) in the intercropping and monoculture fields. IC and MC indicate the center lines (inoculation line) in intercropping and monoculture fields. Significant differences are based on ANOVA tests (P < 0.05). The error bars indicate the standard errors of the means (n = 3).


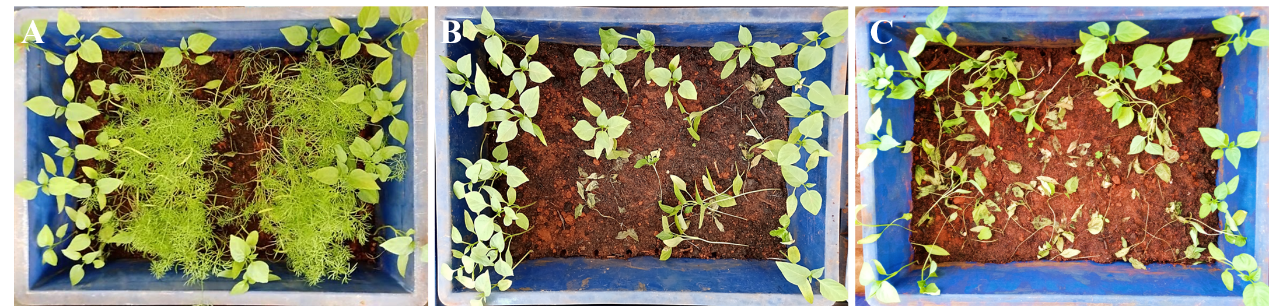


**FIGURE S2|** The disease effect diagram of Fennel and pepper. (A) Pepper and fennel intercropping; (B) Pepper/blank intercropping; (C) Pepper monoculture;
